# Crowdsourcing for Food Purchase Receipt Annotation via Amazon Mechanical Turk: A Feasibility Study

**DOI:** 10.2196/12047

**Published:** 2019-04-05

**Authors:** Wenhua Lu, Alexandra Guttentag, Brian Elbel, Kamila Kiszko, Courtney Abrams, Thomas R Kirchner

**Affiliations:** 1 Department of Childhood Studies Rutgers, The State University of New Jersey Camden, NJ United States; 2 College of Global Public Health New York University New York, NY United States; 3 School of Medicine New York University New York, NY United States; 4 Robert F Wagner Graduate School of Public Service New York University New York, NY United States

**Keywords:** Amazon Mechanical Turk, food purchase receipt, crowdsourcing, feasibility, reliability, validity

## Abstract

**Background:**

The decisions that individuals make about the food and beverage products they purchase and consume directly influence their energy intake and dietary quality and may lead to excess weight gain and obesity. However, gathering and interpreting data on food and beverage purchase patterns can be difficult. Leveraging novel sources of data on food and beverage purchase behavior can provide us with a more objective understanding of food consumption behaviors.

**Objective:**

Food and beverage purchase receipts often include time-stamped location information, which, when associated with product purchase details, can provide a useful behavioral measurement tool. The purpose of this study was to assess the feasibility, reliability, and validity of processing data from fast-food restaurant receipts using crowdsourcing via Amazon Mechanical Turk (MTurk).

**Methods:**

Between 2013 and 2014, receipts (N=12,165) from consumer purchases were collected at 60 different locations of five fast-food restaurant chains in New Jersey and New York City, USA (ie, Burger King, KFC, McDonald’s, Subway, and Wendy’s). Data containing the restaurant name, location, receipt ID, food items purchased, price, and other information were manually entered into an MS Access database and checked for accuracy by a second reviewer; this was considered the *gold standard*. To assess the feasibility of coding receipt data via MTurk, a prototype set of receipts (N=196) was selected. For each receipt, 5 turkers were asked to (1) identify the receipt identifier and the name of the restaurant and (2) indicate whether a beverage was listed in the receipt; if yes, they were to categorize the beverage as cold (eg, soda or energy drink) or hot (eg, coffee or tea). Interturker agreement for specific questions (eg, restaurant name and beverage inclusion) and agreement between turker consensus responses and the gold standard values in the manually entered dataset were calculated.

**Results:**

Among the 196 receipts completed by turkers, the interturker agreement was 100% (196/196) for restaurant names (eg, Burger King, McDonald’s, and Subway), 98.5% (193/196) for beverage inclusion (ie, hot, cold, or none), 92.3% (181/196) for types of hot beverage (eg, hot coffee or hot tea), and 87.2% (171/196) for types of cold beverage (eg, Coke or bottled water). When compared with the gold standard data, the agreement level was 100% (196/196) for restaurant name, 99.5% (195/196) for beverage inclusion, and 99.5% (195/196) for beverage types.

**Conclusions:**

Our findings indicated high interrater agreement for questions across difficulty levels (eg, single- vs binary- vs multiple-choice items). Compared with traditional methods for coding receipt data, MTurk can produce excellent-quality data in a lower-cost, more time-efficient manner.

## Introduction

The decisions that individuals make about the food and beverage products they purchase and consume directly influence their energy intake and dietary quality and may lead to excess weight gain and obesity [1-3]. Research supports the notion that decision making related to food consumption may act as a potential mediator between the neighborhood food environment and individual dietary intake [4-7], but assessment of dietary behavior can be problematic [1]. Leveraging new sources of data on food and beverage purchase behavior, therefore, could provide novel insights into food and beverage decision making.

Food purchase receipts contain information about all foods and beverages purchased by individuals and households from different sources, such as fast-food restaurants, grocery stores, and convenience or corner stores [1]. Compared with retrospective self-reports, receipts can contribute more objective data, thereby avoiding social desirability influence and recall bias [8]. Unfortunately, accurately and reliably annotating large numbers of receipts and images has been a logistical bottleneck inhibiting their widespread use. Typically, academic researchers depend on undergraduate and graduate research assistants to extract data; research progress then depends on the ebb and flow of the semester. Further, each receipt must be carefully reviewed, which takes several minutes. As a result, it can take weeks, months, or even years to process receipt data, especially when large datasets are being handled and/or subjective reasoning is needed.

In the past decade, crowdsourcing has become increasingly popular due to its time-saving and cost-effective qualities [9]. In crowdsourcing, potentially large jobs are broken into many microtasks that are then outsourced directly to individual workers via public solicitation [10]. As the leading and most well-established online crowdsourcing service, Amazon Mechanical Turk (MTurk) enables researchers and businesses, identified as requesters, to recruit anonymous online workers (ie, turkers) worldwide to complete Human Intelligence Tasks (HITs) (ie, tasks that cannot be entirely automated and require human intelligence) at relatively low cost [11]. MTurk offers a basic user interface for simple tasks and a powerful application programming interface for developers to build a platform that uses their services [10,11].

Since its inception, MTurk has been used primarily by researchers in nonmedical fields (eg, psychology, marketing, management, business, political science, computer science, and neuroscience) to do data processing, including data extraction, transcription, translation, and sentiment analysis [12-18]. Emerging studies in recent years have also applied MTurk in various disciplines of health [12-14]. For example, a group of researchers pioneered the use of crowdsourcing technology in public health research and utilized a custom MTurk interface for analyzing mobile phone photographs of retail point-of-sale tobacco marketing [19,20]. Over the course of one typical implementation, 299 turkers completed more than 23,000 tasks at a total cost of US $2500 in less than 24 hours. Results of the crowdsourced photo-only assessments had an excellent level of correspondence to the traditional field survey data, which demonstrated the tremendous potential and reliability of MTurk as a medium for analyzing health-related data in a low-cost, time-efficient way [19,20].

Despite its growing popularity, MTurk has not yet been used to annotate data from food and beverage purchase receipts. This study, therefore, takes an initial foray into assessing the feasibility, reliability, and validity of processing fast-food restaurant receipt data using MTurk.

## Methods

### Overview

Our study consisted of three phases: First, data from a large number of food and beverage purchase receipts were obtained through a traditional in-laboratory, manual data extraction method and were confirmed for accuracy to serve as the *gold standard*; second, an MTurk project was set up and a group of turkers were recruited to extract some prespecified required data from a representative sample of the receipts; and third, the data processed through MTurk were compared with the gold standard and evaluated for reliability and validity. Details of each step are described in the following sections.

### Step 1: Data Collection and Manual Data Extraction

Between August 2013 and May 2014, receipts were collected from consumer purchases at 60 different locations of five fast-food restaurant chains in New Jersey and New York City, USA: Burger King, KFC, McDonald’s, Subway, and Wendy’s. Data collectors stood outside of the restaurants and asked entering customers to save their receipts; upon leaving, they were asked to hand over their receipt and identify which items on the receipt they purchased for their own consumption. Altogether, three rounds of data collection were conducted within the project period, and a total of 12,165 receipts were collected. Detailed data collection procedure, the number of locations surveyed, and receipts collected by location and restaurant chain have been described previously [21].

**Figure 1:**
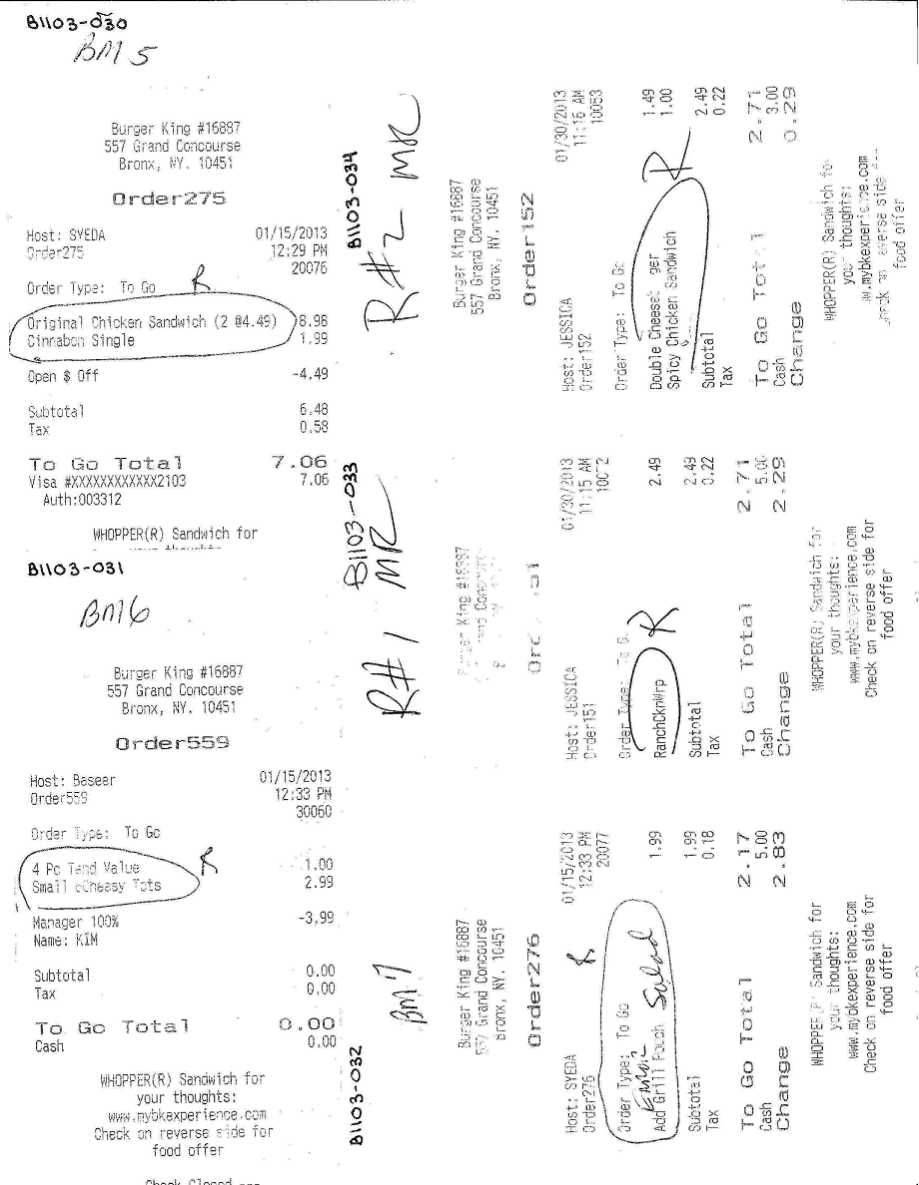
Sample food purchase receipts.

After each round of receipt collection, research assistants pasted receipts on single printer paper sheets next to each other—about 4-6 per page—and scanned them into a database (see Figure 1). Unusual receipts were flagged and reported in the database; for example, those where details of exactly what was purchased by the customers were missing (ie, the receipt was not clearly marked, the ink rubbed off, or the receipt was not itemized).

Following that, the data containing the receipt identifier, restaurant name, food items purchased, purchase date, total price, etc, were extracted from individual receipts; manually entered into an MS Access database by a research assistant; and checked for accuracy by a second research assistant 1-10 months following the initial data entry. The receipt data obtained through such a traditional in-laboratory manual data extraction method served as the gold standard in this study.

### Step 2: Setting Up the Amazon Mechanical Turk Task and Crowdsourcing Workflow

To assess the feasibility of MTurk as an alternative tool for processing receipt data, some of the data extracted manually above was recollected using crowdsourcing via MTurk.

Specifically, two separate tasks were set up on MTurk. The first was an *expert* task, in which turkers were asked to crop one receipt per page at a time from the original pages with 4-6 receipts per page. An *expert* task in MTurk means that requesters trust one turker to do the assignment, rather than having multiple turkers do it and agree on an answer; such tasks are usually simple and do not involve extensive human reasoning and interpretation. First, scanned PDF documents (8.5 x 11 inches) with multiple receipts in different orientations on each page were uploaded onto MTurk. Following that, the MTurk Expert HIT was launched and turkers were recruited to crop each individual receipt using a crop tool and orient the receipts in a readable fashion. An instructional video was included to guide turkers in using the software correctly. This task was completed preceding this study for all receipts (N=12,165), with one receipt on one page.

**Figure 2:**
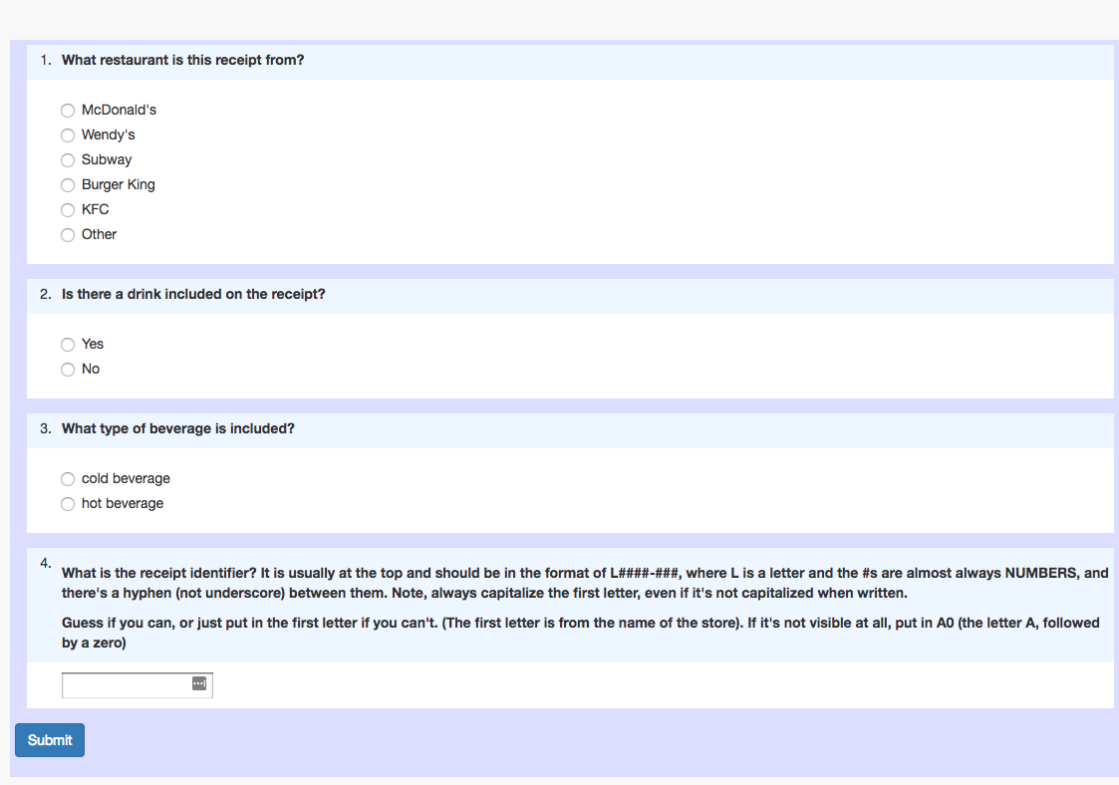
Screenshot of the Amazon Mechanical Turk consensus task.

The next MTurk HIT was a *consensus* task—the focus of this study—which required multiple turkers working on the same assignment and then checking the agreement among their responses. For this task, entitled “Receipt Information,” turkers were requested to identify required information from the food purchase receipts and respond to a series of questions based on the information they identified. As illustrated in Figure 2, a brief description was included under the title of the project: “Please gather the following information related to food purchase from a receipt.” For each receipt, turkers were asked to answer questions based on the following four tasks: (1) write down the receipt identifier, (2) choose the name of the restaurant from a drop-down list, (3) indicate whether a beverage was listed in the receipt, and (4) if a beverage was listed, categorize the beverage as cold beverage or hot beverage.

Specifically, question 1 required textual responses; for each individual receipt, turkers were requested to type in a unique identifier composed of letters and numbers (eg, B1103-036 and S2109A-022). Questions 2-4 included multiple-choice questions, which required subjective judgment at different difficulty levels (ie, single- vs binary-choice items). Considering that most information on food purchase receipts can be obtained through either textual responses or multiple-choice questions, it is reasonable to assume that if a turker can understand and respond accurately to these four exemplary questions, he or she could identify other data from food purchase receipts as well. For demonstration purposes, therefore, instead of using all of the 12,165 receipts, a prototypical sample of receipts (N=196) were used for this study, all of which were clearly marked with zero or only one beverage item on each receipt.

After the prototypical receipts were selected and the MTurk HIT was set up, we started to invite turkers to work on the consensus task. To avoid spammers and control the quality of turkers, we screened the turkers by setting the minimum prior approval rating to 99%, meaning that at least 99% of a turker’s answers to the MTurk HITs that they have completed to date were approved by the requester. Turkers’ locations were further restricted to the United States, as previous studies have suggested that language, cultural background, and ethnicity can significantly influence people’s comprehension of culture-related information such as food choices [16,22,23]. Turkers were paid US $0.06 for interpreting each receipt, which was anticipated to take 60-90 seconds. Once a turker began processing a receipt, he or she had a maximum of 5 minutes to complete it. A turker could analyze as many receipts as he or she wanted.

One critical question in using crowdsourcing via MTurk is setting the minimum and maximum number of turkers who will complete each assignment (ie, the number of repetitions that each receipt will receive until consensus is achieved for each question in the study). Intuitively, 2 would be the absolute minimum in order to reach an agreement on responses to a question. However, setting 2 as the minimum number of repetitions can incur an incorrect agreed-upon answer if both turkers provide the same incorrect responses to a question. A minimum of 3 creates a majority, but the question agreement threshold (QAT) of 67% (2/3) is insufficiently low and an incorrect consensus can still be reached if 2 of the 3 turkers agree on an incorrect answer. A minimum of 4 is acceptable with an interturker agreement of 75% (3/4), but a consensus cannot be achieved if 2 turkers agree on one response while the other 2 turkers agree on another response.

Thus, to reach an agreement with high accuracy, a minimum of 5 turkers is required with a QAT of 60% (3/5). The maximum number of repetitions should also be set as 5, because after starting with a QAT of 60%, the probability of getting an interturker agreement of 80% will then decrease and it will unnecessarily delay the item consensus calculation process if continuing to add more turkers to complete the task. In this study, therefore, 5 was set as both the minimum and maximum number of repetitions that each receipt received with a QAT of 80% (4/5), meaning that a receipt would continue to be available for turkers to interpret until it was assessed 5 times by 5 turkers and at least 4/5 (80%) turkers agreed on a response.

Before the formal launch of the MTurk tasks, several trial runs were performed using small subsets of pictures (ie, 10-20 receipt images at a time) to confirm that the tasks would be manageable by turkers and to confirm that the questions were easily understood and completed. While doing assignments, turkers could reach out to the requester directly by email or through the *Report a Problem with this Task* tab on the MTurk survey interface. During the trial tasks, we received several email inquiries from turkers regarding uncertainties about numbers versus letters in receipt identifiers, receipt number cutoff from the image at the top, etc. No turker expressed problems with interpreting other receipt information, either during the trial runs or the formal task.

### Step 3: Evaluating the Reliability and Validity of Amazon Mechanical Turk

After all assignments were completed, we started evaluating the reliability and validity of MTurk for processing food purchase receipt information. This was conducted in three steps. First, interturker agreement was examined on responses to the four questions asked (ie, receipt identifier, restaurant name, beverage included or not, and type of beverage), and a majority response for each individual question was identified. Following that, turkers’ majority responses were compared with gold standard values in the manually entered dataset to evaluate the reliability and validity of MTurk. Finally, we conducted sensitivity testing to assess whether and how the number of turkers completing each assignment would influence the agreement between turkers’ majority responses and the gold standard. All analyses were conducted using R version 3.2.4 (R Foundation) [24].

## Results

In total, 209 turkers participated in the *consensus* task and initiated or attempted 1346 assignments, among which 983 (73.03%) were approved or completed. On average, each turker contributed 4.7 assignments (SD 1.5). It took an average of 93.12 seconds (SD 70.5, median 65.0) for a turker to analyze a receipt; the entire project was completed within 40 minutes after we launched it on MTurk, with a total cost of US $80.80.

Table 1 lists the descriptive characteristics of the 196 prototypical receipts completed by 5 turkers. Among the 196 receipts that we sampled, one beverage item was listed in 140 receipts (71.4%), including 101 cold beverages (51.5%) and 39 hot beverages (19.9%). Among the 101 receipts with cold beverages, soda drinks were listed in 75 receipts (74.3%), including Coca-Cola, Sprite, Pepsi, Diet Coke, and generic drinks. The rest of the cold-beverage receipts included sweet tea (6/26, 23%), bottled water (5/26, 19%), milkshake or smoothie (5/26, 19%), iced coffee or coffee drinks (4/26, 15%), lemonade (3/26, 12%), and juice or juice beverages (3/26, 12%). Among the 39 receipts with hot beverages, hot coffee was listed in 36 receipts (92%), and the other 3 receipts included 1 with hot chocolate (3%) and 2 with hot tea (5%).

**Table 1:** Descriptive characteristics of the prototypical sample of receipts (N=196).

Restaurant	Number of receipts, n (%)	Number of receipts with beverages out of all receipts from the restaurant, n (%)	Number of hot beverages^a^ out of all receipts from the restaurant, n (%)	Number of cold beverages^b^ out of all receipts from the restaurant, n (%)
Burger King	15 (7.7)	12 (80)	0 (0)	12 (80)
KFC	40 (20.4)	29 (73)	8 (20)	21 (53)
McDonald’s	102 (52.0)	78 (76.5)	31 (30.4)	47 (46.1)
Subway	21 (10.7)	5 (24)	0 (0)	5 (24)
Wendy’s	18 (9.2)	16 (89)	0 (0)	16 (89)

^a^Hot beverages included hot coffee, hot chocolate, and hot tea.

^b^Cold beverages were mostly soda drinks, sweet tea, bottled water, and coffee drinks.

Turkers showed high agreement on their responses to the four questions that we asked. Specifically, among the 196 receipts that we sampled, the proportion of receipts with a QAT of at least 80% (ie, 4/5 interturker agreement) was 100% (196/196) for receipt identifier, 100% (196/196) for restaurant names (eg, Burger King, McDonald’s, or Subway), 98.5% (193/196) for beverage inclusion (ie, yes or no), 92.3% (181/196) for hot beverage (eg, hot coffee or hot tea), and 87.2% (171/196) for cold beverage (eg, soda or bottled water). At a QAT of 100%, the proportions of receipts with unanimous (ie, 5/5) agreement among the turkers was 100% (196/196) for receipt identifier, 90.8% (178/196) for restaurant names, 75.5% (148/196) for beverage inclusion, 69.4% (136/196) for hot beverages, and 51.0% (100/196) for cold beverages.

We further checked the disagreement pattern among turkers for specific questions. For the two questions on receipt identifiers and restaurant names, no disagreement was observed among turkers. When asked to indicate whether a beverage was included or not, disagreements started to emerge. For some cases, turkers overlooked beverages, especially soda drinks that were included in a combo rather than being listed as separate items. For others, some turkers wrongly categorized receipts with smoothies as *beverage not included*. Consequently, when it came to coding the specific type of beverage (ie, cold or hot beverage), more discrepancies were noted.

When comparing turkers’ majority responses with the gold standard data, the agreement rate was 100% (196/196) for receipt identifier, 100% (196/196) for restaurant name, 99.5% (195/196) for beverage inclusion, and 99.5% (195/196) for beverage types. We further tested whether and how the number of turkers influenced the agreement level between turkers’ majority responses and the gold standard data. Based on the analysis, when 3 turkers completed the project, the agreement between their consensus response and the gold standard data was 100% (196/196) for receipt identifier, 100% (196/196) for restaurant name, 99.5% (195/196) for beverage inclusion, and 99.5% (195/196) for beverage type, which were the same as the proportions when 5 turkers completed the assignments.

## Discussion

This study is the first effort to assess whether MTurk, a popular crowdsourcing platform, can be used for processing data from food purchase receipts. In general, findings from this study supported the feasibility, reliability, and validity of MTurk as a cost-effective and time-efficient tool for processing food purchase receipt data.

Findings from this study demonstrated that, with minimal training, the MTurk workforce can categorize and analyze receipt data in a timely and cost-effective way. Despite the low compensation rate (ie, US $0.06 for every assignment), turkers in this study completed the entire task in less than 40 minutes, and the data extracted were of excellent quality, which was consistent with evidence from previous evaluation studies [9,13,18,25,26]. In fact, turkers in previous studies have expressed other motivations that enticed them to complete tasks. For example, many turkers felt it was a productive way to spend available free time, was mentally engaging, was oftentimes interesting, and offered a source of entertainment [27-30]. Compared with manual data extraction, which is often time-consuming, expensive, and difficult to scale up, MTurk can greatly enhance the widespread use of receipts as an assessment of food purchase and dietary behaviors.

Our findings further supported the reliability and validity of using MTurk for annotating receipt data, with high interrater agreement for both textual and multiple-choice questions. Previous studies have noted that as data coding tasks became more subjectively difficult, it got harder to achieve interpretive convergence [26]. Consistently in this study, we found perfect agreement (ie, 100%) among 5 turkers for the two easier questions that did not require subjective judgement (ie, receipt identifier and restaurant name), but we found increased disagreements for the two questions regarding beverage inclusion and beverage type. Nevertheless, when turkers’ majority responses were compared with the manually extracted gold standard, perfect or close-to-perfect agreements were observed, which confirmed the reliability of the number of 5 turkers that we requested for annotating individual receipts.

Our study has limitations. First, although we purposely selected receipts with clearly identifiable information, receipts used in health data analysis could sometimes be more difficult to read due to rips, pen markings, or small font, which would likely affect the agreement rates of turkers. Second, we only allowed turkers with prior approval ratings of 99% to participate in the task. Although this helped to ensure that turkers provide quality work, it also narrowed down the number of turkers available and likely increased the time for task completion. We did not test whether or how lowering the approval rating would affect the reliability and validity of data processing. Third, for demonstration purposes, the tasks we selected were objectively easy; future studies are warranted to determine if the same success rates can be obtained with more complicated tasks.

Despite the limitations, findings from this study hold important practical and research implications. First, our work confirmed the feasibility and accuracy of using MTurk as an innovative approach for processing data from food purchase receipts. In the future, the traditional model of manually annotating food purchase receipts as the gold standard for comparison may be flipped. Instead, crowdsourcing platforms could be used with appropriate task qualification requirements (eg, requiring turkers with prior approval ratings of 95% or 99%) to identify majority or consensus responses, followed by manually annotating a proportion of the receipts to confirm the reliability and validity. This feasibility study demonstrates the scalable and sustainable nature of this approach. Second, the accuracy of crowdsourced receipt annotation in this study lends strong support to the appropriateness of the number of turkers that we requested for each task. To get reliable consensus or majority responses among turkers when annotating image data on MTurk, we recommend future researchers set 5 as both the minimum and maximum number of repetitions for each image, with a question agreement threshold of 80%. Lastly, and most importantly, findings from this study point to the great potential of crowdsourcing for processing data in public health research, particularly tasks that cannot be entirely automated by computer programs and require human intelligence. A recent study has confirmed that objectively documented household food purchases from receipts can yield an unbiased and reasonably accurate estimate of overall diet quality as measured through 24-hour diet recalls [31]. With its time-saving and cost-effective qualities, crowdsourcing will vastly increase capacity for large-scale and high-quality receipt annotation, which in turn will advance our understanding of environmental influence on human health behaviors and ultimately lead to better health prevention and intervention efforts.

## Abbreviations

HIT: Human Intelligence Task
MTurk: Amazon Mechanical Turk
QAT: question agreement threshold

## References

[1] French SA, Shimotsu ST, Wall M, Gerlach AF (2008). Capturing the spectrum of household food and beverage purchasing behavior: A review. J Am Diet Assoc.

[2] Drewnowski A, Rehm CD (2013). Energy intakes of US children and adults by food purchase location and by specific food source. Nutr J.

[3] (2016). National Restaurant Association.

[4] French SA, Wall M, Mitchell NR, Shimotsu ST, Welsh E (2009). Annotated receipts capture household food purchases from a broad range of sources. Int J Behav Nutr Phys Act.

[5] Gordon-Larsen P (2014). Food availability/convenience and obesity. Adv Nutr.

[6] Laska MN, Hearst MO, Forsyth A, Pasch KE, Lytle L (2010). Neighbourhood food environments: Are they associated with adolescent dietary intake, food purchases and weight status?. Public Health Nutr.

[7] White M (2007). Food access and obesity. Obes Rev.

[8] Elbel B, Kersh R, Brescoll VL, Dixon LB (2009). Calorie labeling and food choices: A first look at the effects on low-income people in New York City. Health Aff (Millwood).

[9] Ranard BL, Ha YP, Meisel ZF, Asch DA, Hill SS, Becker LB, Seymour AK, Merchant RM (2014). Crowdsourcing: Harnessing the masses to advance health and medicine, a systematic review. J Gen Intern Med.

[10] Mason W, Watts DJ (2009). Financial incentives and the "performance of crowds". SIGKDD Explor.

[11] Amazon Mechanical Turk.

[12] Khare R, Good BM, Leaman R, Su AI, Lu Z (2016). Crowdsourcing in biomedicine: Challenges and opportunities. Brief Bioinform.

[13] Créquit P, Mansouri G, Benchoufi M, Vivot A, Ravaud P (2018). Mapping of crowdsourcing in health: Systematic review. J Med Internet Res.

[14] Bohannon J (2016). Psychology: Mechanical Turk upends social sciences. Science.

[15] Saunders DR, Bex PJ, Woods RL (2013). Crowdsourcing a normative natural language dataset: A comparison of Amazon Mechanical Turk and in-lab data collection. J Med Internet Res.

[16] Yu B, Willis M, Sun P, Wang J (2013). Crowdsourcing participatory evaluation of medical pictograms using Amazon Mechanical Turk. J Med Internet Res.

[17] Leroy G, Endicott JE, Kauchak D, Mouradi O, Just M (2013). User evaluation of the effects of a text simplification algorithm using term familiarity on perception, understanding, learning, and information retention. J Med Internet Res.

[18] Kuang J, Argo L, Stoddard G, Bray BE, Zeng-Treitler Q (2015). Assessing pictograph recognition: A comparison of crowdsourcing and traditional survey approaches. J Med Internet Res.

[19] Brabham DC, Ribisl KM, Kirchner TR, Bernhardt JM (2014). Crowdsourcing applications for public health. Am J Prev Med.

[20] Ilakkuvan V, Tacelosky M, Ivey KC, Pearson JL, Cantrell J, Vallone DM, Abrams DB, Kirchner TR (2014). Cameras for public health surveillance: A methods protocol for crowdsourced annotation of point-of-sale photographs. JMIR Res Protoc.

[21] Cantor J, Torres A, Abrams C, Elbel B (2015). Five years later: Awareness of New York City's calorie labels declined, with no changes in calories purchased. Health Aff (Millwood).

[22] Dowse R, Ehlers MS (2001). The evaluation of pharmaceutical pictograms in a low-literate South African population. Patient Educ Couns.

[23] Kim H, Nakamura C, Zeng-Treitler Q (2009). Assessment of pictographs developed through a participatory design process using an online survey tool. J Med Internet Res.

[24] R Project.

[25] Azzam T, Jacobson MR (2013). Finding a comparison group. Am J Eval.

[26] Mitra T, Hutto C, Gilbert E (2015). Comparing person- and process-centric strategies for obtaining quality data on Amazon Mechanical Turk. Proceedings of the 33rd Annual ACM Conference on Human Factors in Computing Systems.

[27] Snow R, O'Connor B, Jurafsky D, Ng A (2008). Cheap and fast: But is it good? Evaluating non-expert annotations for natural language tasks. Proceedings of the 2008 Conference on Empirical Methods in Natural Language Processing.

[28] Chandler D, Kapelner A (2013). Breaking monotony with meaning: Motivation in crowdsourcing markets. J Econ Behav Organ.

[29] Horton JJ, Rand DG, Zeckhauser RJ (2011). The online laboratory: Conducting experiments in a real labor market. Exp Econ.

[30] Paolacci G, Chandler J, Ipeirotis P (2010). Running experiments on Amazon Mechanical Turk. Judgm Decis Mak.

[31] Appelhans BM, French SA, Tangney CC, Powell LM, Wang Y (2017). To what extent do food purchases reflect shoppers' diet quality and nutrient intake?. Int J Behav Nutr Phys Act.

